# Isocitrate Dehydrogenase from *Streptococcus mutans*: Biochemical Properties and Evaluation of a Putative Phosphorylation Site at Ser102

**DOI:** 10.1371/journal.pone.0058918

**Published:** 2013-03-06

**Authors:** Peng Wang, Ping Song, Mingming Jin, Guoping Zhu

**Affiliations:** Key Laboratory of Molecular Evolution and Biodiversity and Institute of Molecular Biology and Biotechnology, College of Life Sciences, Anhui Normal University, Wuhu, Anhui, China; University of Leeds, United Kingdom

## Abstract

Isocitrate deyhdrogenase (IDH) is a reversible enzyme in the tricarboxylic acid cycle that catalyzes the NAD(P)^+^-dependent oxidative decarboxylation of isocitrate to α-ketoglutarate (αKG) and the NAD(P)H/CO_2_-dependent reductive carboxylation of αKG to isocitrate. The IDH gene from *Streptococcus mutans* was fused with the *icd* gene promoter from *Escherichia coli* to initiate its expression in the glutamate auxotrophic strain *E. coli Δicd::kan^r^* of which the *icd* gene has been replaced by kanamycin resistance gene. The expression of *S. mutans* IDH (SmIDH) may restore the wild-type phenotype of the *icd*-defective strain on minimal medium without glutamate. The molecular weight of SmIDH was estimated to be 70 kDa by gel filtration chromatography, suggesting a homodimeric structure. SmIDH was divalent cation-dependent and Mn^2+^ was found to be the most effective cation. The optimal pH of SmIDH was 7.8 and the maximum activity was around 45°C. SmIDH was completely NAD^+^ dependent and its apparent *K*_m_ for NAD^+^ was 137 μM. In order to evaluate the role of the putative phosphorylation site at Ser102 in catalysis, two “stably phosphorylated” mutants were constructed by converting Ser102 into Glu102 or Asp102 in SmIDH to mimick a constitutively phosphorylated state. Meanwhile, the functional roles of another four amino acids (threonine, glycine, alanine and tyrosine) containing variant size of side chains were investigated. The replacement of Asp102 or Glu102 totally inactivated the enzyme, while the S102T, S102G, S102A and S102Y mutants decreased the affinity to isocitrate and only retained 16.0%, 2.8%, 3.3% and 1.1% of the original activity, respectively. These results reveal that Ser102 plays important role in substrate binding and is required for the enzyme function. Also, Ser102 in SmIDH is a potential phosphorylation site, indicating that the ancient NAD-dependent IDHs might be the underlying origin of “phosphorylation mechanism” used by their bacterial NADP-dependent homologs.

## Introduction

Isocitrate deyhdrogenase (IDH) catalyzes the NAD(P)^+^-dependent oxidative decarboxylation of isocitrate to α-ketoglutarate (αKG) and the NAD(P)H/CO_2_- dependent reductive carboxylation of αKG to isocitrate using NAD^+^ (EC 1.1.1.41) or NADP^+^ (EC 1.1.1.42) as a cofactor. Phylogenetic analysis reveals that NAD^+^ use by IDH is an ancestral phenotype and NADP^+^ use by prokaryotic IDH arose on or about the time that eukaryotic mitochondria first appeared, some 3.5 billion years ago, in order to synthesize NADPH for bacterial adaptation on acetate [1]. As a better phenotype acquired through evolution, most IDHs exhibit the NADP^+^ dependence, causing NAD^+^ dependence comparatively uncommon among the bacterial IDHs. A small number of NAD^+^-IDHs have been characterized in some bacteria and archaea [2]–[7]. A general feature shared by these organisms is that they have an incomplete tricarboxylic acid (TCA) cycle due to the absence of one or more TCA cycle enzymes [3]. Thus, these NAD^+^-IDHs have been proposed to be reminiscent of the enzyme that participates in CO_2_ fixation as well as glutamate biosynthesis [2], [8]. Eukaryotic NAD^+^-IDHs localize exclusively in the mitochondria and play a central catabolic role in energy production. The enzyme is structural complex and is rate-limiting in the TCA cycle as its affinity for substrate is allosterically regulated by ADP [9].

Eukaryotes have several types of NADP^+^-dependent IDH isoenzymes, distributed in mitochondrial matrix, cell cytosol and peroxisome, respectively [10], [11]. These enzymes share low sequence identity with prokaryotic counterparts, typified by NADP^+^-IDH from *Escherichia coli* (EcIDH). Eukaryotic NADP^+^-IDHs constitute a single clade in the phylogenetic tree, suggesting that they have evolved independently [5]. Mitochondrial isoenzymes provide auxiliary source of α-ketoglutarate and mitochondrial NADPH while the other two isoenzymes function in cellular defense against oxidative damage, detoxification of reactive oxygen species, and providing reducing power and carbon skeleton for fatty acids and amino acids biosynthesis [12]–[15]. Human cytosolic NADP^+^-IDH1 has recently been reported to be involved in tumorigenesis [16]–[18]. The IDH1 Arg132 mutation impairs the oxidative IDH activity of the enzyme, but acquires a new reduction function of converting α-ketoglutarate to 2-hydroxyglutarate, the resulting 2-hydroxyglutarate accumulation then induces the formation and malignant progression of tumors [19], [20].

The prokaryotic NADP^+^-IDHs have been extensively studied. EcIDH lies at the critical juncture between TCA cycle and the glyoxylate bypass, a pathway needed for growth on non-fermentative carbon sources such as acetate and ethanol. Under these stressful conditions, ∼75% of EcIDH is completely inactivated by phosphorylation at Ser113 catalyzed by the bifunctional IDH kinase/phosphatase (IDH K/P), thereby partitioning most of the isocitrate through the glyoxylate shunt [21]. Briefly, isocitrate is hydrogen-bonded to the γ-hydroxyl of Ser113 in the active, dephosphorylated enzyme. The transfer of the γ-phosphate from ATP to Ser113 prevents isocitrate binding by eliminating this hydrogen bond and by introducing a source of electrostatic repulsion and steric hindrance with the γ-carboxylate of isocitrate [22]–[25]. This active serine in substrate binding is highly conserved in both prokaryotic and eukaryotic IDHs and the putative phosphorylation role of the equivalent serine in several NADP^+^-IDHs have been discussed [10], [11], [26]–[28]. Although the NADP^+^-IDH of *Bacillus subtilis* (BsIDH) is not regulated by phosphorylation *in vivo* due to the absence of the gene encoding IDH K/P in this organism, it serves as the substrate for *E. coli* IDH K/P *in vitro*, and the phosphorylation does occur at the expected serine and inhibits IDH activity [27]. Despite the high conservation of the analogous serine in NAD^+^-IDHs, no studies have been reported yet to evaluate the possibility of the phosphorylation regulatory mechanism in these NAD^+^-IDHs.

The NAD^+^-dependent IDH of *S. mutans* (SmIDH) has a Ser102 at the position equivalent to Ser113 of EcIDH. In order to elucidate the function of Ser102, we generated two “stably phosphorylated” (pseudophosphorylated) mutants of SmIDH by converting Ser102 into glutamate or aspirate [29], [30]. Meanwhile, another four mutations at site 102 were analyzed. These mutated enzymes were firstly screened by phenotypic complementation of *icd* deletion strain *E. coli Δicd::kan^r^* and then purified to homogeneity. The kinetic parameters of the wild-type together with the mutated enzymes were determined.

## Materials and Methods

### Strains, media and reagents

The *E. coli* strain *Δicd::kan^r^* was presented by Antony M. Dean's laboratory (BioTechnology Institute, University of Minnesota, MN 55108, USA), which was constructed by replacing the entire *icd* cistron of *E. coli* strain CGSC6300 with a kanamycin cassette [1]. In our study, this auxotrophic strain was used as the host strain for the expression of SmIDH and its mutants. LB and MD media were prepared and supplemented with 100 μg/ml ampicillin and/or 30 μg/ml kanamycin as required. Plates contain 15 g/L agar. PrimeStar^TM^ HS DNA polymerase was obtained from TaKaRa (Dalian, China). Restriction enzymes and protein molecular weight standards were purchased from Fermentas (Shanghai, China).

### Plasmid construction

The 800-bp upstream region of the *icd* gene in the wildtype *E. coli* strain CGSC6300 was amplified with the following primers: *S-Ec^icdp^*, 5′-atagatacctcgagCCATTGGCAAGATTATCCAAAGAGT-3′ (*Xho*I site (underlined letters) and additional bases are indicated by lowercase letters); *R-Ec^icdp^*, 5′-CCCTTCTTCAAAACTTACTTTTTCTGCCATTCACCTCTCCTTCGAGCGCTACTGGTTTGC-3′, which contains the promoter sequence of *E. coli icd* gene. The *S. mutans citC* gene was amplified with the following primers: *S-Sm^icd^*, 5′-GCAAACCAGTAGCG CTCGAAGGAGAGGTGAATGGCAGAAAAAGTAAGTTTTGAAGAAGGG-3′; *R-Sm^icd^*, 5′-tatattctctgcagCTAGTGGTGGTGGTGGTGGTGTAAATAAGTCAATAGAAC-3′ (*Pst*I site (underlined letters) and additional bases are indicated by lowercase letters) using plasmid pKM49 [31] as template. The 3′ end of *R-Ec^icdp^* and the 5′ end of *S-Sm^icd^* were designed to be reverse and complementary to each other so that the two PCR products can be fused together by overlap extension using *S-Ec^icdp^* and *R-Sm^icd^* as primers. The resulting 2.0-kb PCR product containing *S. mutans citC* gene preceded by the promoter of *E. coli icd* gene was then ligated into *Xho*I- and *Pst*I-digested pSP72 (Promega) to create pWT.

### Site-directed mutagenesis

All mutants (S102T, S102G, S102A, S102Y, S102D and S102E) were constructed by site-directed mutagenesis from pWT. The synthetic oligonucleotide primers are shown in Table 1. The mutations were introduced by sequential steps of PCR. In the first round, two reactions (I and II) were performed with the following primers: *S-Ec^icdp^* and one of the antisense primers containing the desired mutation (reaction I); one of the sense primers containing the desired mutation and *R-Sm^icd^* (reaction II). The purified two overlapping fragments were used as templates in the final amplification step with primers *S-Ec^icdp^* and *R-Sm^icd^*. All the final PCR products were cloned into pSP72 to obtain plasmids pS102T, pS102G, pS102A, pS102Y, pS102D and pS102E. All mutated genes were confirmed by sequencing in both directions.

**Table 1:** Construction of SmIDH mutants.

Enzyme	Amino acid sequence a	PCR	
		Template	Primer b
SmIDH	GIRSLNVALRQE		
S102T	- - - T - - - - - - - -	pWT	GGTATTCGTACTTTAAATGTTGCCCTGCGTCAAGAA
S102G	- - - G - - - - - - - -	pWT	GGTATTCGTGGCTTAAATGTTGCCCTGCGTCAAGAA
S102A	- - - A - - - - - - - -	pWT	GGTATTCGTGCTTTAAATGTTGCCCTGCGTCAAGAA
S102Y	- - - Y - - - - - - - -	pWT	GGTATTCGTTATTTAAATGTTGCCCTGCGTCAAGAA
S102D	- - - D - - - - - - - -	pWT	GGTATTCGTGATTTAAATGTTGCCCTGCGTCAAGAA
S102E	- - - E - - - - - - - -	pWT	GGTATTCGTGAATTAAATGTTGCCCTGCGTCAAGAA

a Dashes indicate the same amino acid residues as SmIDH.

b Only sense primers are shown. Underlines indicate mutated regions.

### Protein expression and purification

All recombinant plasmids were transformed into *E. coli* strain *Δicd::kan^r^*, respectively. The resulting strains were grown overnight at 37°C in LB medium with 100 μg/ml ampicillin, and then inoculated (1∶50) into 500 ml of MD medium with the same antibiotic and grown for two days. The cells were harvested by centrifugation, resuspended in wash buffer (10 mM KH_2_PO_4_ (pH 7.7), 500 mM NaCl, 2 mM MgCl_2_ and 2 mM β-mercaptoethanol) and disrupted by sonication. Then, cell debris was removed by centrifugation at 11,000 rpm for 10 min. The 6His-tagged wild-type SmIDH and its mutated enzymes were purified using BD TALON Metal Affinity Resin (Clontech, LaJolla, CA, USA) according to the manufacturer's instructions.

### Enzyme purity and western blotting

Enzyme purity was determined by SDS-PAGE. For western blotting analysis, SDS-PAGE gels were transferred to nitrocellulose membranes by electroblotting and blocked for 1 h at room temperature in TBS-T (50 mM Tris-HCl pH 7.5, 150 mM NaCl, 0.2% Tween-20) containing 5% nonfat milk and then washed with TBS-T for three times. His-tag polyclonal antibody (Cell Signaling Technology Inc., Beverly, MA, USA) was applied to the blots for 1 h at room temperature. After three 10-min washes with TBS-T, the blots were incubated for 1 h with alkaline phosphatase conjugated anti-rabbit IgG (Promega, Madison, WI, USA ). The blots were washed three times in TBS-T, and bound conjugate was revealed by incubation with the alkaline phosphatase substrate. The chemiluminescence signal corresponding to the specific antibody-antigen reaction was visualized by exposing the blots to X-ray film for 15 minutes in the dark room.

### Gel filtration chromatography

The molecular mass of SmIDH was estimated by gel filtration chromatography on a HiLoad^TM^ 10/300 Superdex 200 column (Amersham Biosciences), equilibrated with 0.05 M potassium phosphate buffer (pH 7.0) containing 0.15 M NaCl and 0.01% NaN_3_. Protein standards for calibrating gels were Ovalbumin (45 kDa), Conalbumin (75 kDa), Aldolase (158 kDa), Ferritin (440 kDa) and Thyroglobulin (669 kDa).

### Circular dichroism spectroscopy of the wild-type and mutant enzymes

Circular dichroism (CD) spectroscopy was conducted using a Jasco model J-810 spectropolarimeter. The ellipticity measurements as a function of wavelength were performed as described previously [32]. Purified protein samples (0.3 mg/ml) were prepared in 50 mM sodium phosphate and 60 mM NaCl (pH 7.5). The ellipticity (θ) was obtained by averaging 3 scans of the enzyme solution between 200 and 260 nm at 0.5 nm increments. The mean molar ellipticity, [*θ*] (deg cm^2^ dmole^−1^), was calculated from [*θ*]  =  *θ*/10*nCl*, where *θ* is the measured ellipticity (millidegrees), *C* is the molar concentration of protein, *l* is the cell path length in centimeters (0.1 cm), and *n* is the number of residues per subunit of enzyme (399 for SmIDH and the mutants).

### Enzyme assays and kinetic studies

The enzyme activity was assayed by a modification of the method described previously [33]. Reaction mixtures were incubated at 37°C in 1 ml volume containing 35 mM Tris-HCl buffer (pH 7.5), 3.5 mM MnCl2, 2.5 mM DL-isocitrate, 0.5 mM NAD^+^ or 5 mM NADP^+^. The increase in NADPH or NADH was monitored at 340 nm with a thermostated Cary 300 UV-Vis spectrophotometer (Varian, CA, USA) using a molar extinction coefficient of 6220 M^−1^ cm^−1^. One unit (U) of activity was defined as 1 μmol NADPH or NADH formed per minute. The apparent kinetic parameters were calculated by nonlinear regression using the program Prism 5.0 (Prism, GraphPad Software, CA, USA). All kinetic parameters were obtained from at least three measurements. The concentrations of the purified enzymes were estimated by absorbance measurements at 280 nm, using an extinction coefficient (ε_280_ = 51,255) calculated by the method of Pace et al. [34].

### Effects of pH and temperature

The enzyme was assayed in 35 mM Tris-HCl buffer between pH 7.2 and 9.2. The optimum temperature was determined by the standard activity assay at different temperatures from 25°C to 55°C. To estimate thermal stability, enzymes were incubated for 20 min between 25 and 55°C in a water bath. Aliquots were withdrawn at periodic intervals, cooled in an ice bath and assayed as described above.

### Metal ions effect

The effects of different metal ions (2 mM MnCl_2_, 2 mM MgCl_2_, 2 mM CaCl_2_, 2 mM CoCl_2_, 2 mM CuCl_2_, 2 mM ZnSO_4_, 2 mM NiSO_4_, 2 mM NaCl and 2 mM KCl) on SmIDH activities were determined using the standard assay procedures.

### Structure-based amino acid sequence alignment

X-ray structures of *E. coli* NADP-IDH (EcIDH, PDB code 9ICD), *Bacillus subtilis* NADP-IDH (BsIDH, 1HQS) and *Acidithiobacillus thiooxidans* NAD-IDH (AtIDH, 2D4V) were downloaded from the PDB database (http://www.rcsb.org/pdb/). The homology model of SmIDH was generated by SWISS-MODEL server (http://swissmodel.expasy.org). Structure-based amino acid sequence alignment was conducted with ClustalX program (ftp://ftp.ebi.ac.uk/pub/software/clustalw2) and ESPript 2.2 web tool (http://espript.ibcp.fr/ESPript/ESPript/) [35], [36].

## Results and Discussion

### Expression and purification of recombinant SmIDH

As the recombinant SmIDH can not be produced in *E. coli* under the control of the *lac* promoter, a unique IDH expression method was applied in this study. The promoter sequence of *E. coli icd* gene was firstly fused to the N-terminus of *S. mutans citC* gene and then subcloned together into the vector pSP72, creating pWT. The recombinant plasmid pWT was transformed into the glutamate auxotrophic *E. coli* strain *Δicd::kan^r^* (*icd*-defective strain). The expression of *S. mutans citC* gene was examined by evaluating the strain growth on minimal medium containing glucose as the sole carbon source without glutamate. The transformants that grew well on the minimal medium were isolated, indicating that *S. mutans citC* gene can be expressed normally and function well by restoring the growth of *E. coli* strain *Δicd::kan^r^*.

**Figure 1:**
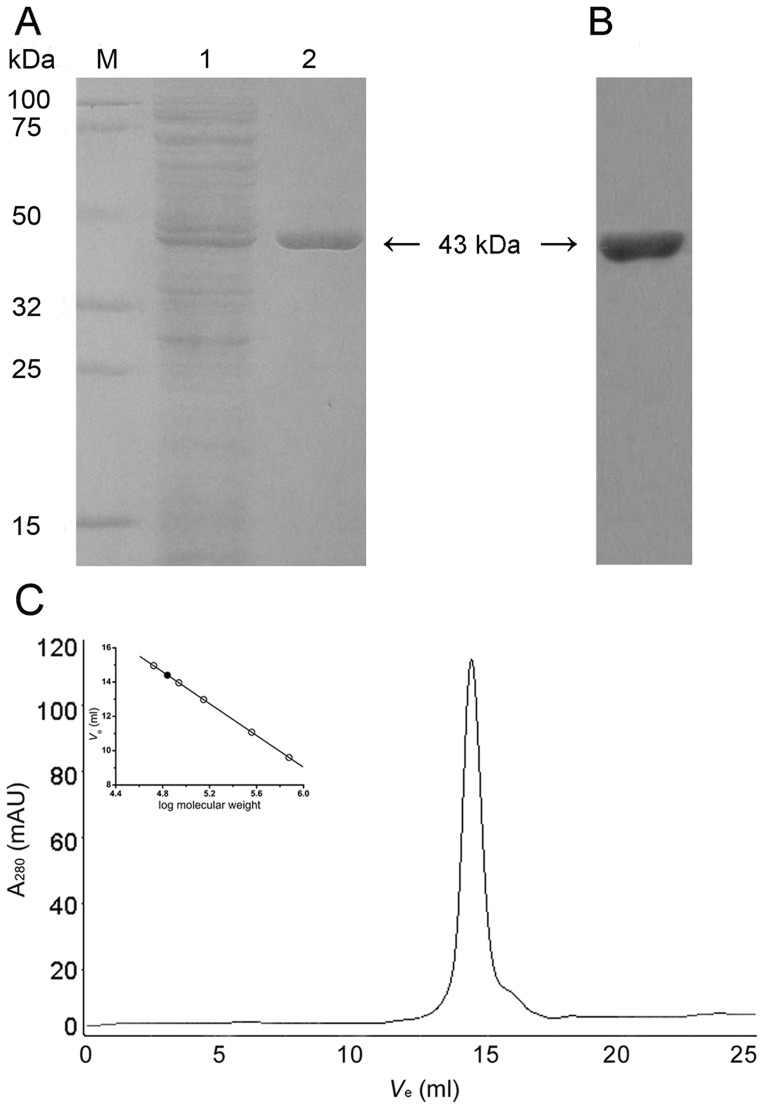
Enzyme purity, western blot and molecular mass analysis of SmIDH. (A) SDS-PAGE analysis of the expression and purification of SmIDH. Analysis was performed on 12% polyacrylamide gel. M, protein markers; lane 1, crude extracts of cells harboring pWT grown in MD medium without glutamate. lane 2, purified protein. (B) Western blot analysis using anti-6×His antibody as probe. (C) Molecular mass determination by gel filtration chromatography. The flow rate was 0.5 ml/min and the proteins in the fractions were monitored at 280 nm. Inside is the standard curve for molecular mass. SmIDH was represented as a dark circle (•). Standard proteins were represented by open circles (○): 1, Ovalbumin (44 kDa); 2, Conalbumin (75 kDa); 3, Aldolase (158 kDa); 4, Ferritin (440 kDa); 5, Thyroglobulin (669 kDa). *V*_e_ of SmIDH is 14.4 ml.

SmIDH was produced as a 6-histidine fusion protein that had a calculated molecular mass of 43 kDa. A single band at around 43 kDa was visible on the SDS-PAGE gel (Figure 1A), which was confirmed by Western blotting using anti-His antibody (Figure 1B). Gel filtration chromatography was performed to determinate the oligomerization status of SmIDH in solution. The native molecular mass of SmIDH estimated by gel filtration was 70 kDa (Figure 1C), suggesting a homodimeric structure similar to *E. coli* IDH, *B. subtilis* IDH and *A. thiooxidans* IDH [8], [26], [37].

### Characterization of the enzymatic properties of SmIDH

**Figure 2:**
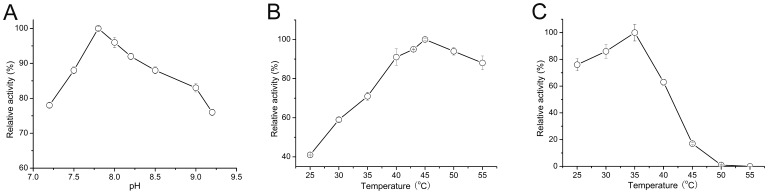
Effects of pH and temperature on the activity of purified SmIDH. (A) Effects of pH on SmIDH activity was measured with the pH range of 7.2–9.2. (B) Effects of temperature on SmIDH activity was measured from 25°C to 55°C. (C) Heat-inactivation profiles of SmIDH. The enzyme activity was measured from 25°C to 55°C.

The effects of pH on SmIDH activity were performed in the pH range of 7.2–9.2. SmIDH was found to have an optimal pH range of 7.5–8.5, with the optimal pH at 7.8 (Figure 2A), lower than that of the NAD^+^-IDHs from *A*. *thiooxidans* (pH 8.5) [2] and *Hydrogenobacter thermophilus* (pH 10.5) [38]. When compared with the broad optimum pH range of NADP^+^-IDHs from other sources, such as *Streptomyces lividans* (pH 8.5–10.0) [39], *Fomitopsis palustri*s (pH 8.0–10.0) [40] and *Aspergillus niger* (pH 6.0–8.0) [41], it was narrower for SmIDH, suggesting that SmIDH was sensitive to pH changes.

SmIDH had a temperature optimum for activity at 40–53°C and its maximum activity was observed at 45°C (Figure 2B). Although this mesophilic enzyme is stable at room temperature, 50% loss of activity and almost complete inactivation were observed after incubation at 43°C and 50°C for 20 min, respectively (Figure 2C). This data was quite different from some known NAD^+^-IDHs while most of them were found to be thermostable, such as *A*. *thiooxidans* IDH (stable up to 55°C) [2], *Methylococcus capsulatus* IDH (optimum for activity at 55–60°C) [4], *H. thermophilus* IDH (half-inactivation at 88.7°C) [3] and most distinguished *P. furiosus* IDH (with a melting temperature of 103.7°C) [5]. The significant difference in thermostability among NAD^+^-IDHs could be the temperature adaptation of enzymes to their niches.

**Figure 3:**
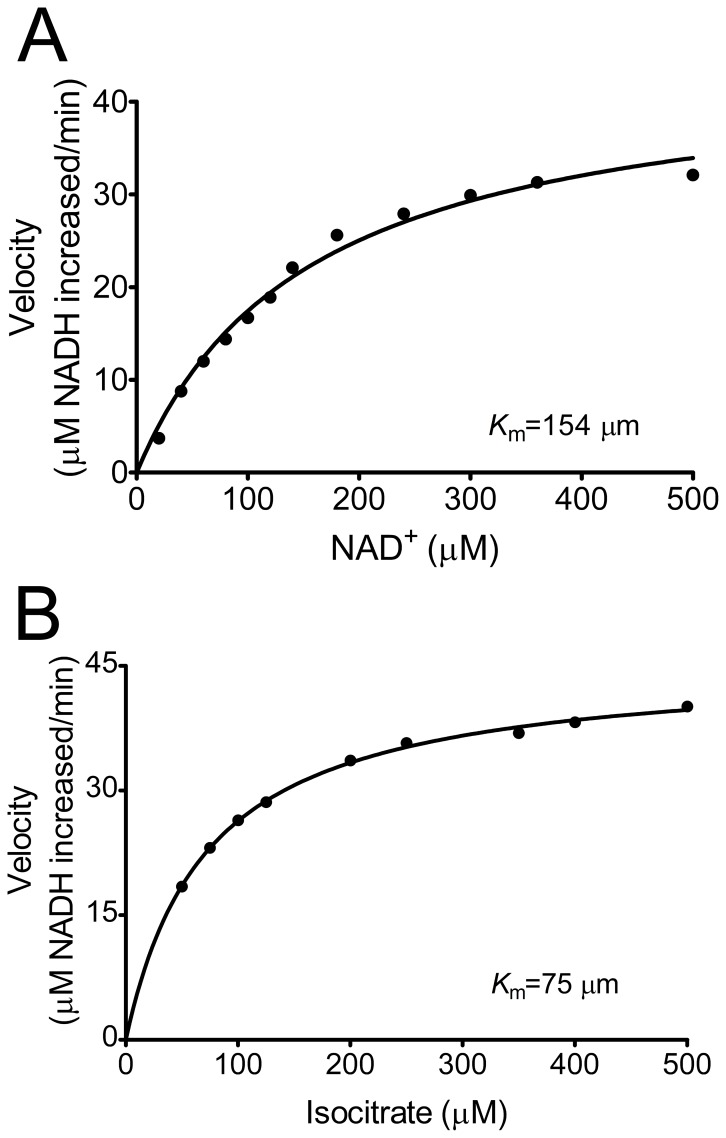
Kinetic analysis of the recombinant SmIDH. The kinetic parameters of the recombinant SmIDH were determined by measuring its enzyme activity at various isocitrate or NAD^+^ concentrations with the other substrate at saturating concentrations. Enzymatic activity was assessed by monitoring the increase of NADH. The SmIDH *K*_m_ for NAD^+^ (A) and isocitrate (B) were calculated as 154 μM and 75 μM, respectively, by averaging values from triplicate experiments.

**Figure 4:**
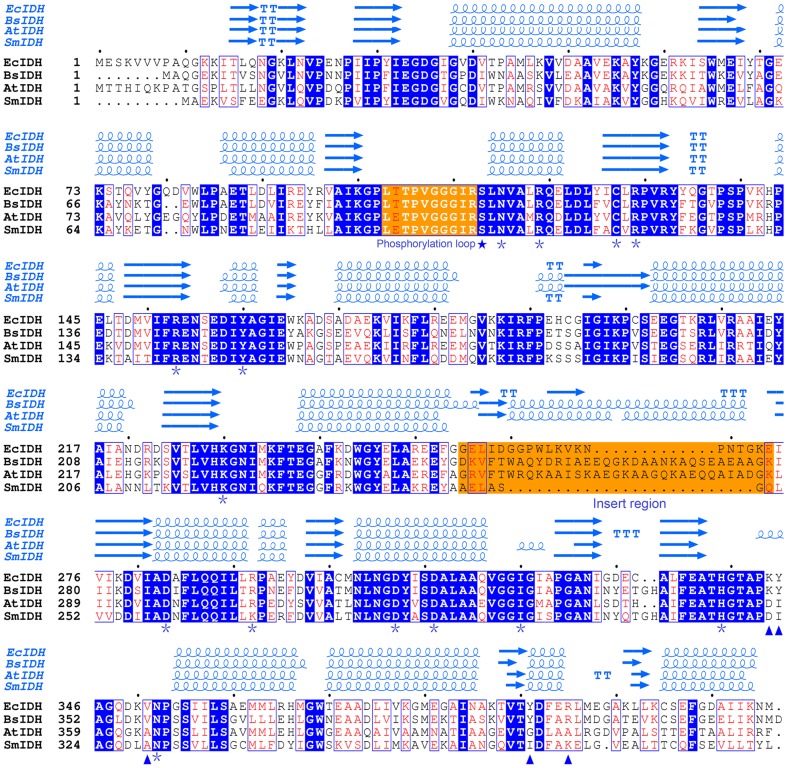
Structure-based protein sequence alignment of SmIDH with other dimeric IDHs. High-resolution crystal structures of *E. coli* NADP-IDH (EcIDH, 9ICD), *B. subtilis* NADP-IDH (BsIDH, 1HQS) and *A. thiooxidans* NAD-IDH (AtIDH, 2D4V) were downloaded from the PDB database. SmIDH structure was generated using the SWISS-MODEL modeling server, using AtIDH as a template structure. Invariant residues are highlighted by shaded blue boxes and conserved residues by open blue boxes. The conserved residues involved in the cofactor binding (▴) and substrate binding (*) are indicated. The phosphorylation site was represented by ★. The phosphorylation loop and the insert region were highlighted by shaded orange boxes. The figure was made with ESPript 2.2 [36].

Kinetic studies revealed that the apparent *K*_m_ of SmIDH displayed for NAD^+^ and isocitrate was 154 μM and 75 μM, respectively (Figure 3). The *K*_m_ value of SmIDH for NAD^+^ was higher than those determined for *P. furiosus* IDH (68.3 μM) and *M. capsulatusbut* IDH (122 μM), but lower than that observed for NAD^+^-IDHs from *A. thiooxidans* (180 μM), *Streptococcus suis* (233 μM), *Zymomonas mobilis* (312 μM) and *H. thermophilus* IDH (357 μM) [2]–[7]. SmIDH was completely NAD^+^ dependent as shown and no enzyme activity was observed for SmIDH when NAD^+^ was substituted by NADP^+^ in concentrations up to 5 mM. The cofactor discrimination of IDH was determined by only a few amino acids [42]. In some NADP^+^-IDHs, such as EcIDH and BsIDH, Lys^344/350^ and Tyr^345/351^ are the major NADP^+^-specificity determinants (Figure 4), which are substituted by the conserved Asp and Ile in all known NAD^+^-IDHs such as Asp^322/357^ and Ile^323/358^ in SmIDH and AtIDH (Figure 4). These major specificity determinants can be used as reliable landmarks to predict the coenzyme specificity of new IDHs.

The effects of nine different metal ions on SmIDH activity were examined (Table 2). SmIDH activity was entirely dependent on the presence of a divalent cation, and Mn^2+^ was found to be the most favored one although Mg^2+^ can partially replace it. In the presence of Mn^2+^, the addition of 2 mM Co^2+^, Cu^2+^, Zn^2+^ and Ni^2+^ reduced SmIDH activity to about 11%, 12%, 2% and 1% of control value, respectively (Table 2). However, the dramatic inhibition of Ca^2+^ on SmIDH activity was not observed in our study. This was quite different from some reports on NADP^+^-IDHs, such as *E. coli* IDH and *S. lividans* IDH, that Ca^2+^ may reduce the activity at a large scale [39], [43]. Neither Na^+^ nor K^+^ affected SmIDH activity in the presence of Mn^2+^.

**Table 2:** Effects of metal ions on the activity of recombinant SmIDH a.

Metal ions	Relative activity (%)
None	0
K^+^	15±1.7
Na^+^	23±2.1
Mn^2+^	100±0.5
Mg^2+^	84±2.3
Co^2+^	7±0.3
Cu^2+^	1±0.1
Ca^2+^	0
Zn^2+^	0
Ni^2+^	0
K^+^+Mn^2+^	108±5.8
Na^+^+Mn^2+^	101±4.9
Mg^2+^+Mn^2+^	84±2.0
Co^2+^+Mn^2+^	11±1.2
Cu^2+^+Mn^2+^	12±1.4
Ca^2+^+Mn^2+^	81±3.6
Zn^2+^+Mn^2+^	2±0.7
Ni^2+^+Mn^2+^	1±0.3

a The values indicate the means of at least three independent measurements.

### Performances of Ser102 mutants

IDH is the first bacterial enzyme shown to be regulated by phosphorylation/dephosphorylation [44]. The modulation of IDH activity enables *E. coli* to make rapid shifts between TCA and glyoxalate bypass pathways, whereas the phosphorylation state of IDH determines its activity [45]–[47]. Phosphorylation on a serine residue of EcIDH by IDH K/P inactivates the enzyme by preventing NADP^+^ binding, and dephosphorylation reactivates it [44], [46], [48]. Although an analogous serine has been found to be highly conserved in all known NAD^+^-IDHs, which is corresponding to the phosphorylation site of Ser113 in EcIDH, there were no reports on evaluating the role of the analogous serine in the regulatory mechanism of NAD^+^-IDH activity yet.

A putative phosphorylation site Ser102 was present in the NAD^+^-dependent SmIDH. Given the highly structural similarity of SmIDH to EcIDH (Figure 4), the same phosphorylation regulatory mechanism might be used by SmIDH, even though phosphorylation *in vivo* has not been found yet. In this study, we mimicked the phosphorylation state of Ser102 by mutating Ser102 to Asp or Glu (S102D or S102E). The *icd*-defective strain *E. coli Δicd::kan^r^* harboring the recombinant plasmid pS102D or pS102E did not show any growth on MD medium without glutamate, indicating that the mutations of S102D or S102E caused total loss of SmIDH activity. Similar results were reported by Thorsness et al. [24] and Matsuno et al. [31], and they found that both EcIDH and BsIDH were nearly abolished by replacing Ser with Asp in the active site. Given the possibility that a small amount of activity can be retained by S102D and S102E mutants, as observed in S113D and S113E mutants of EcIDH in the previous studies [22], [23], [25], [49], the residual activities of the mutants were so low that the *icd*-defective *E. coli* was not able to survive on MD plates without glutamate.

Phylogenetic analysis reveals that NAD^+^ use by IDH is an ancestral phenotype and NADP^+^ use by prokaryotic IDH arose on or about the time that eukaryotic mitochondria first appeared, some 3.5 billion years ago. The switch of the coenzyme specificity of prokaryotic IDH from NAD^+^ to NADP^+^ is an ancient adaptation to anabolic demand for NADPH during growth on acetate [1]. The anaerobic, Gram-positive bacterium *S. mutans* has an IDH with ancient NAD^+^-dependency, suggesting that *S. mutans* might be an ancient prokaryote and not be selected by poor carbon sources (i.e. two carbon compounds) through its evolutionary history. Mimicking phosphorylation by replacing Ser102 with Asp or Glu inactivates SmIDH, implying that Ser102 is a potential phosphorylation site and the ancient NAD-dependent IDHs might be the underlying origin of “phosphorylation mechanism” used by their bacterial NADP-dependent homologs.

**Figure 5:**
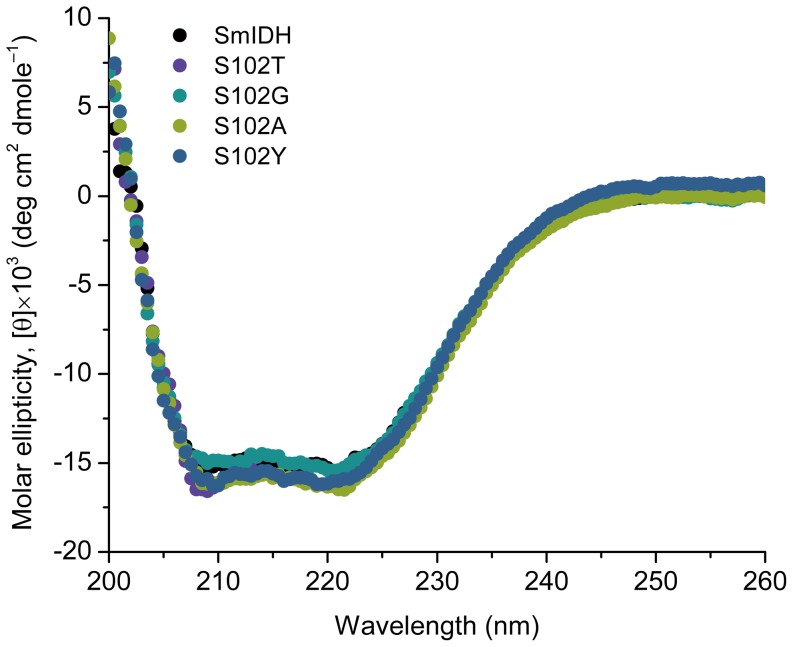
Circular diochroism (CD) spectra of the wild-type SmIDH and four mutants, S102T, S102G, S102A and S102Y. The CD was measured and the molar ellipticity was calculated as described in Materials and methods.

**Figure 6:**
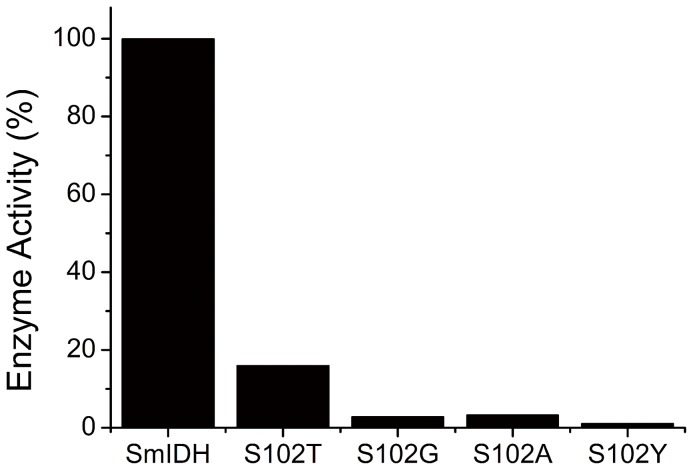
The residual activities of the four Ser102 mutants as compared to the wild-type SmIDH.

The effects of amino acids with different size of side chains, such as Thr, Gly, Ala and Tyr, at the site 102 were also investigated. *E. coli Δicd::kan^r^* strains containing pS102T, pS102G, pS102A and pS102Y showed different growth performances in minimal media, respectively, and the four mutant enzymes were then purified to homogeneity. CD spectra of the wild-type and mutant SmIDH were measured to evaluate whether there is any change in secondary structure of these mutant enzymes. The CD spectra of S102T, S102G, S102A, and S102Y mutants are very similar to that of the wild-type enzyme (Figure 5), suggesting that the mutations did not cause any appreciable conformational change. Four mutants decreased the affinity to isocitrate with 2- to 5-fold *K*_m_ values of the wild-type enzyme. Consequently, the catalytic efficiency (*k*_cat_/*K*_m_) of them was remarkably reduced about 10- to 412-fold, and S102T, S102G, S102A and S102Y only retained 16.0%, 2.8%, 3.3% and 1.1% of the original activity, respectively (Figure 6). In S102T mutant enzyme, the Thr residue has a γ-hydroxyl side chain as Ser that can bind isocitrate by a hydrogen-bond and thus making S102T retain a relative high activity. The activities of S102Y, S102G and S102A were mainly depended on the size of the side chain of replaced residues, as the bulky side chain in the active site is sterically unfavorable for isocitrate and cofactor binding [22], [25]. For example, the replaced Tyr in S102Y, an amino acid with large side chain, caused almost 99% activity loss of SmIDH (Figure 6). The dramatic loss of S102Y activity was mainly caused by its decreased substrate- and cofactor-binding abilities. As shown in Table 3, the *K*_m_ values for isocitrate and NAD^+^ of S102Y were increased 5.1-fold (from 75 to 385 μM) and 10.1-fold (from 154 to 1560 μM), respectively.

**Table 3:** Kinetic parameters of SmIDH and its mutants for isocitrate and NAD^+^.

Enzyme	Isocitrate			NAD^+^		
	*K*_m_ (μM)	*k*_cat_ (s^−1^)	*k*_cat_/*K*_m_ (μM^−1^ s^−1^)	*K*_m_ (μM)	*k*_cat_ (s^−1^)	*k*_cat_/*K*_m_ (μM^−1^ s^−1^)
SmIDH	75	124	1.65	154	56	0.36
S102T	150	25	0.17	350	59	0.17
S102G	143	4	0.028	295	9.1	0.03
S102A	148	3.3	0.022	246	3	0.01
S102Y	385	1.5	0.004	1560	3.5	0.002

Taken together, six mutations were introduced into SmIDH at Ser102 respectively. Two mutant enzymes lost the activity, and the other four mutants significantly decreased the activity. Apparently, SmIDH activity is sensitive to the substitution at position 102, the conserved Ser102 plays important role in substrate binding and is required for maintaining the proper structure of the active site and responsible for the enzyme function. It implies that SmIDH may have the similar quaternary structure and catalytic mechanism to the well-known NADP-IDHs, such as EcIDH and BsIDH. In addition, the completely conservation of the so-called “phosphorylation loop” and the missing of the “insert-region” (Figure 4), which restricts the access of *E. coli* IDH K/P to the phosphorylation site in BsIDH [26], suggest that SmIDH could be a better substrate for *E. coli* IDH K/P than BsIDH.
